# Supplementary material for: GP-delivered medication review of polypharmacy, deprescribing, and patient priorities in older people with multimorbidity in Irish primary care (SPPiRE Study): A cluster randomised controlled trial
Source: PLoS Med. 2022 Jan 5;19(1):e1003862. doi: 10.1371/journal.pmed.1003862 (PMC8730438; doi:10.1371/journal.pmed.1003862)
Supplement: S1 Text — SPPiRE, Supporting Prescribing in Older Adults with Multimorbidity in Irish Primary Care. (DOCX) [file pmed.1003862.s001.docx]

# S1 SPPiRE study protocol

Published in *Implementation Science* 1^st^ August 2017

**Supporting prescribing in older people with multimorbidity and significant polypharmacy in primary care (SPPiRE): a cluster randomised controlled trial protocol and pilot**

Authors: Caroline McCarthy (carolinemccarthy@rcsi.ie)*^1^, Barbara Clyne (barbaraclyne@rcsi.ie)^1^, Derek Corrigan (derrickcorigan@rcsi.ie)^1^, Fiona Boland (fionaboland@rcsi.ie)^1^, Emma Wallace (emmawallace@rcsi.ie)^1^, Frank Moriarty (frankmoriarty@rcsi.ie) ^1^, Tom Fahey (tomfahey@rcsi.ie) ^1^, Carmel Hughes (c.hughes@qub.ac.uk)^2^, Paddy Gillespie (paddy.gillespie@nuigalway.ie)^3^, Susan M Smith (susansmith@rcsi.ie)^1^

*Corresponding author

**^1^** HRB Centre for Primary Care Research, Royal College of Surgeons in Ireland, Beaux Lane House, Lower Mercer Street, Dublin 2, Ireland

^2^ School of Pharmacy, Queen’s University Belfast, 97 Lisburn Road, Belfast BT9 7BL, Northern Ireland

^3^ School of Business & Economics, National University of Ireland Galway, Co. Galway, Ireland

**Abstract**

**Background**

Multimorbidity, defined as the presence of at least two chronic conditions, becomes increasingly common in older people and is associated with poorer health outcomes and significant polypharmacy. The National Institute for Clinical Excellence (NICE) recently published a multimorbidity guideline that advises providing an individualised medication review for all people prescribed 15 or more repeat medicines. This study incorporates this guideline and aims is to assess the effectiveness of a complex intervention designed to support general practitioners (GPs) to reduce potentially inappropriate prescribing and consider deprescribing in older people with multimorbidity and significant polypharmacy in Irish primary care.

**Methods**

This study is a cluster randomised controlled trial, involving 30 general practices and 450 patients throughout Ireland. Practices will recruit older patients, aged 65 years and over, who are prescribed at least 15 repeat medicines and will then be randomised using minimisation to either intervention or control. All GPs will use a software patient finder tool to identify patients through the practice management software system. GPs in intervention practices will receive login details to a website where they will access training videos and a template for conducting an individualised structured medication review, which they will undertake with each of their included patients. The review entails screening the prescription for potentially inappropriate prescribing (PIP) and assessing patient treatment priorities, tailoring treatment accordingly. Control practices will deliver usual care over the six month study period. Primary outcome measures are the proportion of patients with any PIP and the number of repeat medicines.

**Discussion**

Disease-specific approaches in multimorbidity may be inappropriate and result in fragmented and poorly co-ordinated care. This pragmatic study is evaluating a complex intervention that is relevant across multiple conditions and addresses potential concerns around medicines safety in this vulnerable group of patients. The potential for system-wide implementation will be explored with a parallel mixed methods process evaluation.

**Trial registration**

**ISRCTN:** 12752680

Registered 20^th^ October 2016

**Key words**

Complex intervention, Multimorbidity, Polypharmacy, Potentially inappropriate prescribing (PIP) Deprescribing, Patient priorities, Randomised controlled trial

**Background**

Advances in public health and health care have led to increases in life expectancy and a resultant rising population of older people living with multiple long term conditions or multimorbidity, now estimated to be at least 60% for those over 65 years ([1](#_ENREF_1)). There is a growing consensus that the current single disease model of healthcare, which arose out of specialisation, may not be appropriate when managing these patients and may result in fragmented care and significant treatment burden for patients ([2](#_ENREF_2), [3](#_ENREF_3)). Providing optimal care for these patients is challenging, considering their heterogeneity and the lack of evidence for the effectiveness of many treatments, due to exclusion of these patients from randomised controlled trials ([4](#_ENREF_4)). A systematic review looking at interventions to improve outcomes in patients with multimorbidity concluded that targeting risk factors or specific functional difficulties may be most effective ([5](#_ENREF_5)). The recently published National Institute for Health and Care Excellence (NICE) guideline, “Multimorbidity: assessment, prioritisation and management of care for people with commonly occurring multimorbidity” identifies the link between complex multimorbidity and polypharmacy and advises targeting patients who are prescribed ≥ 15 repeat medicines and providing a tailored or individualised structured medication review ([6](#_ENREF_6)).

In line with rising levels of multimorbidity, the prevalence of significant polypharmacy is steadily rising. Recent longitudinal analysis indicates that approximately 5% of Irish people aged over 65 years are prescribed 15 or more repeat medicines ([7](#_ENREF_7)). Polypharmacy is associated with a multitude of adverse outcomes including adverse drug reactions (ADRs), medication errors, reduced adherence, increased cost and increased morbidity and mortality ([4](#_ENREF_4), [8](#_ENREF_8), [9](#_ENREF_9)). ADRs are a significant burden to the health system and it is estimated that over 10% of emergency hospital admissions in older patients may be ADR-related ([10](#_ENREF_10)). ADRs have also been described as the “great mimic of systemic disease” ([11](#_ENREF_11)) and often result in a prescribing cascade, where more medication is used to treat the side effects of another ([12](#_ENREF_12)). In patients with multimorbidity polypharmacy may often be appropriate, however it is the strongest risk factor for potentially inappropriate prescribing (PIP), a term used to describe suboptimal prescribing practices where the risks of treatment usually outweigh the benefits ([13](#_ENREF_13)).

Various tools have been developed to both tackle and measure PIP, including explicit measures of appropriateness such as the United States (US) Beers criteria ([14](#_ENREF_14)) and the European Screening Tool for Older People’s potentially inappropriate Prescriptions (STOPP) criteria ([15](#_ENREF_15)). Explicit measures of medication appropriateness have been demonstrated to be effective at improving prescribing and have the advantage of being relatively reproducible, reliable and easy to apply to large numbers of people ([16](#_ENREF_16)); however they do not account for individual patient characteristics and preferences. Implicit measures such the Medication Appropriateness Index ([17](#_ENREF_17)) involve clinician judgement and do account for patient characteristics, however they are time-consuming and complex to apply, and generally involve a detailed assessment of the effectiveness and appropriateness of each prescribed medication ([4](#_ENREF_4)). Several systematic reviews have assessed various approaches, including the use of explicit and implicit measures of appropriateness, to tackling inappropriate polypharmacy ([18-21](#_ENREF_18)) with the over-riding conclusion that inappropriate polypharmacy is amenable to change but that a multi-faceted approach is likely to be more effective.

Improvements in computerised clinical decision support systems, where individual patient data are inputted into a computer program, sorted and matched using algorithms generated from a prior knowledge base resulting in patient-specific recommendations ([22](#_ENREF_22)), may ultimately prove effective in optimising medication for this complex group of patients. An on-going European Union (EU) funded project is developing a highly powered and efficient software engine that will be able to individually screen the medication of older patients with multimorbidity and provide recommendations on optimal drug therapy and ADR risk ([23](#_ENREF_23)). Similarly Young et al in New Zealand are validating and piloting a set of prescribing decision tools that will be embedded in practice management software (PMS) ([24](#_ENREF_24)). However, before such technology is demonstrated to be safe, effective and feasible in daily practice, it is important to continue to develop effective and pragmatic interventions that can assist GPs in caring and prescribing for these complex patients. We also argue that software driven recommendations need to be accompanied by an assessment of patients’ treatment priorities.

Despite recommendations advising tailoring care to patient priorities, there is very little in the literature on how to best assess and record patient priorities. A systematic review identified only one such tool that has been used and validated in patients with multimorbidity ([25](#_ENREF_25), [26](#_ENREF_26)). A research group in Spain have recently published a protocol for a RCT which will examine the effect of an individualised medication review using a tool designed to aid the physician in prioritising the patient’s problems, on medication appropriateness ([27](#_ENREF_27)). However the effectiveness, feasibility and potential for negative effects of using these tools in practice have not been established.

**Rationale for SPPiRE Trial**

Our research group previously demonstrated that an intervention comprised of academic detailing and a web based prescribing support was effective in reducing potentially inappropriate prescribing in older patients in Irish primary care, primarily through the reduction of prescribing of proton pump inhibitors at maximal dose (OPTI-SCRIPT trial)([34](#_ENREF_34)). Supporting prescribing in older patients with multimorbidity and significant polypharmacy (SPPiRE) is a cluster RCT that will assess the effectiveness of an intervention designed to reduce both PIP and polypharmacy. The SPPiRE intervention evolved based on the OPTI-SCRIPT trial results and process evaluation ([28](#_ENREF_28), [29](#_ENREF_29)), emerging evidence in relation to multimorbidity and polypharmacy ([5](#_ENREF_5), [6](#_ENREF_6), [19](#_ENREF_19), [20](#_ENREF_20), [30](#_ENREF_30)) and the NICE multimorbidity guideline described previously ([6](#_ENREF_6)). In line with the Medical Research Council’s Framework on developing and evaluating complex interventions, the OPTI-SCRIPT intervention and trial were adapted to develop the SPPiRE study (Figure 1). The MRC Framework describes an iterative process where piloting work, process and outcome evaluations and other bodies of emerging evidence feed back into the original intervention design ([31](#_ENREF_31)). The target population and individual components of the intervention have been amended (Table 1). Our proposed SPPiRE intervention will also assess the effect on patient-reported outcome measures (PROMs) of treatment burden and quality of life and undertake a parallel qualitative evaluation, which will provide important evidence on the patient experience.

***<Insert figure 1>***

***<Insert table 1>***

Medication appropriateness will be examined using explicit criteria and a tailored approach, where individual patient priorities are addressed. Tailoring care in this way minimises the harms of overtreatment by prioritising treatments that are most important to the patient ([25](#_ENREF_25), [32](#_ENREF_32)). Explicit criteria have been selected using the updated STOPP 2 criteria ([15](#_ENREF_15)) and monitoring criteria developed and validated by the Data-driven Quality Improvement in Primary care (DQIP) research group ([33](#_ENREF_33)), with a focus on drug classes that are frequently implicated in preventable drug related morbidity (PDRM)([34](#_ENREF_34)) and criteria that were more relevant for older people in Irish primary care (listed in Tables 2 - 4) during the development of our previous OPTI-SCRIPT intervention ([35](#_ENREF_35)). The tool will support GPs to identify PIPs for individual patients and then suggest potential treatment alternatives in the context of patient priorities identified during the process. As described previously several tools have recently been developed to assess patient priorities, however given uncertainty of the effectiveness of these tools, we have opted to prompt the GP to ask about and record individual priorities during the medication review rather than trying to operationalise this process. The implementation and effect of this novel aspect of the intervention will be further explored in the parallel mixed methods process evaluation. Reviewing medicines in the context of both explicit measures of appropriateness and individual patient priorities will ensure that medicines that are potentially inappropriate, either because of ineffectiveness in relation to symptom control, adverse effects or unfavourable risk/benefit ratios are discontinued or changed ([4](#_ENREF_4), [36](#_ENREF_36), [37](#_ENREF_37)).

***<Insert table 2>***

***<Insert table 3>***

***<Insert table 4>***

This paper aims to describe the results of a small scale uncontrolled pilot study of the SPPiRE intervention and the final refinement of the intervention, as well as present the protocol for the definitive randomised controlled trial.

## **Trial Objectives**

The overall aim of this research is to evaluate the effectiveness of a complex intervention (SPPiRE), to support GPs to improve the safety of their prescribing and reduce polypharmacy in patients aged 65 years or over with multimorbidity and significant polypharmacy (15 or more repeat medicines) in Irish primary care.

Specific objectives are:

1. Undertake a definitive cluster randomised controlled trial to evaluate the effectiveness of the multifaceted SPPiRE intervention, comprising professional education and a web-guided structured medication review where patient priorities are assessed, in reducing PIP and polypharmacy.

2. To determine the cost-effectiveness of this intervention.

3. To explore the potential for system-wide implementation of the SPPiRE intervention in Irish primary care through a mixed methods process evaluation.

## **Pilot Study Methods and Results**

## Study design

A small-scale uncontrolled pilot study was undertaken to assess the feasibility and acceptability of the adapted SPPiRE intervention and to assess whether the intervention needed further adaptation ([38](#_ENREF_38)).

*Study Population*

Six general practitioners, including four academic GPs, one full-time clinical GP and one GP registrar piloted the SPPiRE web guided medication review with 10 different patients.

## Procedure

A SPPiRE software patient finder tool was developed to allow GPs to easily identify all their patients aged ≥ 65 years and prescribed ≥ 15 repeat medicines. This tool was piloted by three GPs in three practices, as at the time of the pilot it was only available in one of the two predominant PMS systems used in Ireland. A repeat medicine was defined as any item that has an Anatomical Therapeutic Chemical (ATC) code, is currently being prescribed to the patient and is classified in the PMS as a “repeat” as opposed to “acute” item. GPs also have to manually screen the generated list to ensure “acute” prescriptions have not been inadvertently left on the “repeat” list.

All six GPs piloted the SPPiRE intervention and provided feedback to the trial manager (CMcC) on outcome measures and the feasibility and acceptability of the intervention. Each GP watched the SPPiRE training videos and invited either one or two identified patients for a medication review. The GPs logged onto the SPPiRE website at the start of the medication review and were guided through the medication review process. The steps involved were:

- Counting the current number of repeat medicines
- Screening the current prescription for potentially inappropriate prescriptions using supports provided within the tool
- Assessing patient treatment priorities
- Reviewing each medicine with the patient to assess effectiveness, side-effects and actual drug utilisation
- Agreeing changes with the patient, based on both web-guided suggested alternatives and the patient’s treatment priorities

## Results

The feasibility of the finder tool and intervention were assessed through GP feedback and analysis of use of the online material. Overall the intervention was well received by the GPs and their patients many of whom reported feeling reassured that their medicines were being reviewed and rationalised. There was a large variation between practices in the proportion of those aged ≥ 65 years detected by the finder tool (1.9%- 17%) which may be partially explained by differences between practices in how prescription lists are kept up to date and socio-demographic differences, both of which will be identified by the practice profile questionnaire at baseline for the main trial. Due to lack of universal registration of private patients in Irish general practice, practices use varying approaches to count their private patient panel. The differences in the finder tool detection rate may also reflect an inaccurate denominator, where private patients aged ≥ 65 years are over counted for example visitors and those who have moved.

Of the 10 patients who underwent the medication review, they were prescribed an average of 17.5 medicines (SD +/-3.41) and this reduced to 16.8 (SD +/-3.94) immediately post-intervention. All of the included patients had at least one PIP, with a mean of 1.8 per patient. The most common PIP identified were the use of a “proton pump inhibitor for uncomplicated PUD/erosive peptic oesophagitis at full therapeutic dose for ≥ eight weeks” (39%) and the use of a “benzodiazepine or Z drug for longer than four weeks” (39%). When the higher prevalence, lower risk proton pump inhibitor PIP was excluded, 90% of patients had at least one PIP and the mean number of PIP per patient was 1.1. Identified instances of PIP were acted on in 44% of cases and 45% when the proton pump inhibitor PIP was excluded. The most commonly reported patient treatment priorities were treating pain, followed by fatigue and reducing the number of repeat medicines.

*Intervention modification*

Based on the results described above, minor modifications were made to the training videos and the medication review template to make instruction more explicit and reduce repetition (summarised in Table 5). The finder tool was modified to only include items with a unique ATC code to prevent different doses of the same drug being counted twice. The need for the GP to manually screen the generated list was evident and guidance on how to best do this was developed. The review also took longer to implement than anticipated, on average about 40 minutes and a recommendation to allow for this amount time was added to the demonstration video and support materials.

**Protocol for the SPPiRE Trial**

**Methods**

## Study Design

A pragmatic two arm cluster randomised controlled trial will be conducted. The study design was developed in line with the Consolidated Standards of Reporting Trials (CONSORT) statement extension to cluster RCTs ([39](#_ENREF_39)). A cluster design was chosen to avoid the possibility of contamination across arms. If a GP was treating both intervention and control patients, they may find it difficult to behave differently towards each group ([40](#_ENREF_40)). The trial will be conducted in primary care with GP practices as the unit of randomisation. The intervention will be delivered at the GP level while the unit of analysis will be the individual patient, adjusting for clustering. A mixed methods process evaluation and economic analysis will also be conducted.

## Study population

Practices will be eligible to participate if:

- They have at least 300 patients aged ≥65 years on their patient panel. This is based on the need to identify at least 15 patients per practice who are receiving ≥15 repeat medicines (which is 5% of people aged ≥65 years in the national Primary Care Reimbursement Service (PCRS) pharmacy dispensing database ([7](#_ENREF_7))).
- They use Socrates or Health One as a PMS system (to enable use of the software patient finder tool).

Patients will be considered eligible if:

- They are aged ≥65 years.
- They are being prescribed 15 or more repeat medicines, which is a measure of both significant polypharmacy and complex multimorbidity ([41](#_ENREF_41)). A repeat medicine is defined as any item with an ATC code for which the patient is currently in receipt of a prescription of and is classified by the PMS as a “repeat” item.

Practices will be excluded if:

- They are currently involved in a medication management or prescribing trial.

Patients will be excluded if:

- They are unable to give informed consent, as judged by their GP.
- They are unable to attend the practice for a face-to-face medication review.
- They have previously taken part in the OPTI-SCRIPT intervention.

## Recruitment and allocation

Eligible practices (see eligibility criteria above) will be identified through the Health Research Board Primary Care Clinical Trials Network, Ireland (http://primarycaretrials.ie/) and invited to participate by formal letter, including letter of agreement to participate and study information leaflets detailing the steps of the intervention. Practices will identify patients aged 65 years and over who are currently prescribed 15 or more repeat medicines using the patient finder tool, which will be integrated into practice management software in participating practices. The research team will assist practices in recruiting eligible patients, who will be sent invitation letters (on practice headed paper) with consent forms, information leaflets and questionnaires. Based on recent analysis of prescribing trends in Ireland, we anticipate that most included practices will have at least 15 eligible patients ([7](#_ENREF_7)). In larger practices with more than 40 eligible patients, we will randomly select 40 patients to invite, based on the OPTI-SCRIPT response rate ([28](#_ENREF_28)). Control practices will receive €30 per recruited patient and intervention practices €60 per recruited patient to reflect some of the costs involved in taking part in the study.

Baseline data collection will occur prior to practice allocation by review of patient prescriptions and questionnaires (see outcomes and methods for data collection below). Recruited practices will be randomly allocated, using minimisation, by an independent third party (Figure 2). Minimisation will ensure balance between the groups ([39](#_ENREF_39), [40](#_ENREF_40)) in terms of practice size (number of whole-time-equivalent GPs) and location (urban versus rural). An “urban” practice is defined as being located in an area with a population of at least 5,000 people, has most of its patients within a relatively small geographical area, and have ready access some hospital facilities ([42](#_ENREF_42)). Considering the nature of the intervention, it is not possible to blind GPs or participants to the intervention.

***<Insert figure 2>***

*The Intervention*

The content of the SPPiRE intervention was developed based on the OPTI-SCRIPT trial, emerging evidence and results from the SPPiRE pilot and contains the following elements:

1. Training videos which demonstrate how to perform a SPPiRE medication review and describe the underlying evidence on polypharmacy, common potentially inappropriate prescriptions in older people, multimorbidity and treatment burden. The videos also provide guidance on establishing treatment burden and supporting patients to express their priorities, based on recommendations in the NICE multimorbidity guideline ([6](#_ENREF_6)).
2. An online medication review template (see figure 3) which provides a structured process and links to alternative strategies for each identified PIP. The GP will be guided to:
   1. Screen the prescription for PIP, including the requirement for monitoring and if duplicate drug classes are present.
   2. Assess the patient’s treatment priorities and consider whether on-going symptoms may represent adverse drug reactions.
   3. Review each medicine with the patient assessing the effectiveness of symptomatic treatment, the relative harms and benefits of preventative treatment, the presence of adverse drug reactions and whether each medicine is being taken as prescribed.
   4. Agree all changes with the patient, taking into account their treatment priorities and considering suggested treatment alternatives for identified PIP.
   5. Print out a summary document which will be generated upon completion of the review. This will outline all changes and any action that needs to be taken, including the need for blood monitoring if indicated.

Any medication changes will be at the discretion of the prescribing GP, weighing up the risks and benefits and incorporating patient preference.

***<Insert figure 3>***

The control arm will continue to deliver usual care for the six month study duration. Usual care will vary from practice to practice and differences in repeat prescribing policies between clusters will be identified and described using a baseline practice profile questionnaire. Following six-month data collection and completion of the RCT, control arm GPs will be invited to participate in the intervention as outlined above. No further RCT data will be collected from participants on trial completion at six months. However, we will ask intervention practices to submit prescription data at 12 months to examine whether changes in prescriptions have been maintained.

## Contemporaneous National Control – PCRS dataset

We will also analyse national prescription patterns via the Health Service Executive (HSE) primary care reimbursement service (PCRS) prescription database. This is a national prescribing database based on GP and pharmacy claims in a number of community schemes, including the General Medical Services (GMS) scheme. In 2014, 70% of people aged over 65 years in Ireland were covered by the GMS scheme and so their prescription data are captured in the PCRS database ([43](#_ENREF_43)). Anonymised data from the PCRS GMS database can be used to provide a contemporary national control. Comparisons to previous national prescribing patterns for those aged 65 years and over being prescribed 15 or more medicines will determine if there has been a change over time at the population level. ([7](#_ENREF_7))

## Outcomes Measures

The outcomes have been specifically chosen to reflect the potential effects of the intervention and to incorporate the patient perspective. Baseline data will be collected prior to allocation using the PMS, patient questionnaires and a practice profile questionnaire. Follow up data will be collected six months following completion of each medication review in intervention patients through review of the PMS and patient questionnaires and six months following the median date of all the medication review dates in control practices.

*Primary outcomes:*

- Proportion of patients with any PIP (see Tables 2-4)
- Mean number of repeat medications, defined as any medicine with an ATC code for which the patient is currently in receipt of a repeat prescription.

*Secondary outcomes:*

- Reduction in proportion of patients with 15 or more medicines (100% at baseline)
- Medication changes
  - Number of medications stopped (per patient and absolute number in intervention versus control)
  - Numbers of medications started (per patient and absolute number in intervention versus control)
  - Reduction in the number of PIP (per patient and absolute number in intervention versus control)
  - Proportion of patients with any reduction in PIP
- Multimorbidity Treatment Burden Questionnaire (MTBQ)([44](#_ENREF_44))
- Health related Quality of life (EQ5D-5L)([45](#_ENREF_45))
- Revised Patients' attitudes towards deprescribing (PATD)([46](#_ENREF_46))
- Health service utilisation per patient (based on chart data and collected on both time periods (baseline and 6 months post-intervention completion), at study completion by the research team
  - Number of GP/practice nurse visits
  - Number of out-of-hours visits
  - Number of Accident & Emergency (A&E) visits
  - Number of emergency hospital admissions and reason for admission
  - Number of in-patient days
  - Number of out-patient visits
- Patient-reported adverse drug withdrawal events (ADWEs) from follow up patient questionnaire (defined as either recurrence of the condition for which the drug was prescribed or a physiologic reaction to drug withdrawal.) ([47](#_ENREF_47), [48](#_ENREF_48))

## Plan of Analysis

Descriptive statistics (proportions, means) will be used to evaluate differences in baseline characteristics between participating GP practices and patients in the two arms of the trial. The primary analysis will be carried out using multi-level modelling. The primary outcome measure, proportion of patients with or without a PIP, will be a binary outcome measure and will be compared between treatment arms using random effects logistic regression with individuals as the unit of analysis and the practice included as the random effect.

The second primary outcome measure, number of repeat medications, will be assessed using a random effect Poisson regression with the individual as the unit of analysis and the practice included as the random effect to control for the effects of clustering. Incidence rate ratios (IRR) and 95% confidence intervals (CI) will be presented. All analysis will be conducted under the intention-to-treat principle as this is a pragmatic trial ([39](#_ENREF_39)).

## Sample Size

Sample size for the primary outcome (proportion with PIP) is based on a prevalence of approximately 40% for PIP in this population ([7](#_ENREF_7)). To reduce this to 20% requires a sample size of 240 patients. The sample size also needs to be adjusted to reflect the assumption that individual outcomes are not independent of each other, as participants in the same cluster may respond in the same way. Hence, an intra-cluster correlation coefficient (ICC) of 0.025 and 15 patients per practice will be used. This was also used in the OPTI-SCRIPT study and was based on an observational study of an elderly cohort in the HRB Centre for Primary Care Research ([49](#_ENREF_49)). This gives a design effect size of 1.35, thus inflating the sample to 324 patients from 22 practices.

All the patients in this study will be selected on the basis of receiving 15 or more repeat medicines on their prescription. Existing data from the Irish national prescribing database (HSE-PCRS) highlights that in those aged 65 years and over on 15 or more repeat medicines, the mean number of medicines is 17.4 (SD 2.69) ([7](#_ENREF_7)). A meta-analysis of deprescribing trials reported a difference between treatment groups of 0.4 medicines where the mean number of medicines was 7.4 ([50](#_ENREF_50)). We are targeting patients on at least 15 repeat medicines and for primary outcome, number of repeat medications, will test for a mean difference of one medicine between the intervention and control group and this would require a sample size of 306 patients. This is inflated by the design effect size outlined above (1.35) to give a sample of 413 patients from 28 practices.

Factoring in a loss to follow-up of approximately 10% (for primary outcomes, based on chart data) and using the higher numbers required to demonstrate a significant reduction in mean number of repeat medicines, a total of 30 GP practices and 450 patients will be required.

*Process Evaluation*

SPPiRE is a complex intervention as there are several different interacting components and a degree of flexibility or tailoring to the local environment ([31](#_ENREF_31)). The aim of the process evaluation will be to explore the effect of the intervention and how it was implemented. It will combine both quantitative and qualitative data and follow a framework specific for cluster RCTs ([51](#_ENREF_51)).

## Study population

All participating practices and a purposive sample of patients will be invited to participate by letter and follow-up telephone call.

## Process evaluation data collection

Quantitative data will be compiled from a number of sources including completed questionnaires, researcher logs and evaluation forms and website usage data. Qualitative data will be collected from patients and GPs and other practice staff using semi-structured interviews. The number of interviews needed to reach data saturation (where no new themes emerge) will be considered alongside feasibility issues (resources and timing), but a sample of 15-20 is proposed ([45](#_ENREF_45)).

These interviews will be conducted either in person or via telephone. Telephone interviewing is generally used where time or costs are issues, and evidence suggests there is little difference in the answers obtained this way ([52](#_ENREF_52)). All interviews will be audio recorded (on loud speaker for telephone interviews).

*Process evaluation measures:*

- Use of the intervention software
- Clinical/ prescribing decision made e.g. stop or start medicine, refer for monitoring blood test
- Reported primary reason for decision made e.g. risks outweigh benefits, patient preference, hospital/consultant initiated
- Whether assessment of patient priorities resulted in medication change and which priorities were associated with most change
- Immediate pre- and post-intervention prescription

## Process evaluation data analysis

Quantitative data will be summarised using descriptive statistics. All interviews will be transcribed verbatim and broad themes and sub-themes identified using thematic analysis. NVivo 10 (QSR International) will be used to assist with organising the data for analysis.

## Economic Evaluation

The health economic analysis will consist of a trial-based economic evaluation of the proposed intervention and will incorporate both cost effectiveness analysis and cost utility analysis. The evaluation will be undertaken in a manner consistent with guidelines issued by the Health Information and Quality Authority (HIQA) in Ireland ([53](#_ENREF_53)). Evidence collected on resource use, costs, and health outcome measures will provide the basis for the evaluation over the trial follow up period. With respect to costing, a publicly funded health service perspective will be adopted. That is, resource use associated with delivery of the proposed intervention will be measured and costed, as will other health service resource use by patients over the course of the trial. As detailed above, significant attention will be paid to collecting relevant data on health outcomes alongside the trial for the purposes of the clinical effectiveness analysis. For the cost effectiveness analysis, effectiveness will be evaluated on the basis of the number of PIPs averted. For the cost utility analysis, effectiveness will be evaluated in terms of quality adjusted life years (QALYs), which will be estimated based on responses to the EuroQol EQ-5D-5L instrument ([45](#_ENREF_45)). An incremental analysis will be undertaken to provide information on the marginal costs and effects of the intervention relative to the control through the calculation of incremental cost effectiveness ratios (ICERs). The statistical analysis will be conducted in accordance with current guidelines for economic evaluation alongside cluster RCTs ([53](#_ENREF_53)). That is, we will adopt multilevel statistical techniques which recognise both the clustering and correlation in the cost and effect data. Uncertainty in the economic analysis will be addressed by estimating cost effectiveness acceptability curves (CEACs), which link the probability of treatment being cost effective to a range of potential threshold values (λ) that a health system may be willing to pay.

## Data Management & Protection

All patients will provide informed consent and will be known to the research team by study ID only. The patient’s GP remains responsible for all treatment decisions made. Informed consent will be sought from all GPs participating in the study. All data collected, including interview transcripts for the process evaluation, will be stored on a secure, password protected network drive.

## Monitoring and participant safety

A Trial Steering Committee (TSC) comprising an independent chair and two other independent members, one of whom is a lay member representing the patient and public perspective, has been established and will oversee the progress of the trial and adherence to the study protocol. It was agreed by the TSC that given the low risk nature of the trial, lack of interim data and relatively short term follow up, that there is no need for the establishment of a Data Monitoring Committee.

A formal process has been developed to capture any potential adverse events related to discontinuing medication. Recruited practices will be instructed in the practice recruitment leaflet on how to report any suspected adverse effects from deprescribing. Any adverse events will also be captured by a chart review at follow up data collection. An assessment on likely causality will then be made using an adapted drug withdrawal probability scale ([48](#_ENREF_48)). Patient-reported ADWEs will also be collected as a secondary outcome measures in the follow-up patient questionnaire, six months after intervention completion. Any patient safety concerns arising during the course of the trial, including at baseline data collection, will be brought to the attention of the Trial Steering Committee.

**Discussion**

Polypharmacy is frequently cited as an area of major concern by patients with multimorbidity ([54](#_ENREF_54), [55](#_ENREF_55)). The most appropriate course of action when deciding whether to continue to prescribe is often not readily apparent but should include some assessment of the relative harms and benefits of treatment, in the context of the patient’s priorities ([56](#_ENREF_56)). We propose that an individualised structured medication review, where deprescribing is considered alongside prescribing, and where there is active engagement with patients on their views on treatment is essential to tackling polypharmacy and inappropriate prescribing.

The SPPiRE intervention provides structure and assistance in performing medication reviews by aiding GPs in identifying PIP, suggesting alternatives and encouraging care to be tailored to individual patient priorities. Strengths of this study include the pragmatic study design, incorporation of clinical guidelines and the fact that the intervention can be delivered by the patient’s GP. Currently there is no provision under the GP reimbursement scheme in Ireland for a medication review for older patients and the potential and costs of a system-wide implementation of the SPPiRE intervention in Irish primary care will be explored in the process and economic evaluations.

Limitations of the study include the possibility of contamination of the control group where GPs also identify patients on 15 or more medicines using the patient finder tool and may treat these patients differently or provide more consideration than usual when renewing prescriptions. This will be explored in the process evaluation. Patients will also be encouraged to think about their treatment burden and attitudes towards deprescribing when completing baseline questionnaires and may be influenced to discuss their medications with their GP. Secondly, given the nature of the intervention it is not possible to blind patients, GPs or study personnel to the intervention. This has been addressed somewhat by using robust, objective primary outcome measures where there is little room for subjective opinion (number of repeat medicines and number of patients with any pre-defined PIP).

In summary this study will provide evidence on the effectiveness and acceptability of a complex intervention designed to support GPs improve prescribing and reduce polypharmacy for older patients with multimorbidity and significant polypharmacy in Irish primary care.

**List of Abbreviations**

National institute for clinical excellence (NICE), Adverse drug reaction (ADR), Potentially inappropriate prescribing (PIP), European Screening Tool for Older Person’s Prescriptions (STOPP), Medication appropriateness index (MAI), European Union (EU), Medical Research Council (MRC), Patient reported outcome measure (PROM), Randomised controlled trial (RCT), Preventable drug related morbidity (PDRM), National Institute for Clinical Excellence (NICE), General practitioner (GP), Consolidated Standards of Reporting Trials (CONSORT), Primary Care Reimbursement Service (PCRS), Practice management software (PMS), Anatomical Therapeutic Chemical (ATC)

**Declarations**

*Ethics approval and consent to participate*

Full ethical approval for the study was granted by the Irish College of General Practitioners Research Ethics Committee. (ICGP REC; SPPiRE study) Informed consent will be sought from all patients and GPs participating in the study.

*Consent for publication*

Not applicable

*Availability of data and material*

The datasets used and/or analysed during the current study are available from the corresponding author on reasonable request.

*Competing interests*

The authors declare that they have no competing interests.

*Funding*

This research is funded by the Health Research Board (HRB) Primary Care Clinical Trials Network (http://primarycaretrials.ie/). The funding body had no role in the design of the study, the collection, analysis, and interpretation of data or in writing the manuscript.

*Authors' contributions*

CMcC drafted the original manuscript. All authors made contributions to the intervention development and trial design and read and approved the final manuscript.

*Acknowledgements*

Not applicable

# References

1. Fortin M, Stewart M, Poitras ME, Almirall J, Maddocks H. A systematic review of prevalence studies on multimorbidity: toward a more uniform methodology. Annals of family medicine. 2012 Mar-Apr;10(2):142-51.

2. Salisbury C. Multimorbidity: time for action rather than words. The British journal of general practice : the journal of the Royal College of General Practitioners. 2013 Feb;63(607):64-5.

3. Hughes LD, McMurdo ME, Guthrie B. Guidelines for people not for diseases: the challenges of applying UK clinical guidelines to people with multimorbidity. Age and ageing. 2013 Jan;42(1):62-9.

4. Spinewine A, Schmader KE, Barber N, Hughes C, Lapane KL, Swine C, et al. Appropriate prescribing in elderly people: how well can it be measured and optimised? Lancet (London, England). 2007 Jul 14;370(9582):173-84.

5. Smith SM, Wallace E, O'Dowd T, Fortin M. Interventions for improving outcomes in patients with multimorbidity in primary care and community settings. Cochrane Database of Systematic Reviews. 2016 (3).

6. Multimorbidity: assessment, prioritisation and management of care for people with commonly occurring multimorbidity. NICE Guideline. 2016.

7. Moriarty F, Hardy C, Bennett K, Smith SM, Fahey T. Trends and interaction of polypharmacy and potentially inappropriate prescribing in primary care over 15 years in Ireland: a repeated cross-sectional study. BMJ Open. 2015;5(9):e008656.

8. Wallace J, Paauw DS. Appropriate prescribing and important drug interactions in older adults. The Medical clinics of North America. 2015 Mar;99(2):295-310.

9. Guthrie B, McCowan C, Davey P, Simpson CR, Dreischulte T, Barnett K. High risk prescribing in primary care patients particularly vulnerable to adverse drug events: cross sectional population database analysis in Scottish general practice. BMJ. 2011;342:d3514.

10. Kongkaew C, Noyce PR, Ashcroft DM. Hospital admissions associated with adverse drug reactions: a systematic review of prospective observational studies. The Annals of pharmacotherapy. 2008 Jul;42(7):1017-25.

11. Pirmohamed M, Breckenridge AM, Kitteringham NR, Park BK. Adverse drug reactions. BMJ : British Medical Journal. 1998 25th April 1998;316(7140):1295-8.

12. Rochon PA, Gurwitz JH. Optimising drug treatment for elderly people: the prescribing cascade. Bmj. 1997 Oct 25;315(7115):1096-9.

13. Cahir C, Moriarty F, Teljeur C, Fahey T, Bennett K. Potentially inappropriate prescribing and vulnerability and hospitalization in older community-dwelling patients. The Annals of pharmacotherapy. 2014 Dec;48(12):1546-54.

14. American Geriatrics Society 2015 Updated Beers Criteria for Potentially Inappropriate Medication Use in Older Adults. Journal of the American Geriatrics Society. 2015 Nov;63(11):2227-46.

15. O'Mahony D, O'Sullivan D, Byrne S, O'Connor MN, Ryan C, Gallagher P. STOPP/START criteria for potentially inappropriate prescribing in older people: version 2. Age and ageing. 2015 Mar;44(2):213-8.

16. Dimitrow MS, Airaksinen MS, Kivela SL, Lyles A, Leikola SN. Comparison of prescribing criteria to evaluate the appropriateness of drug treatment in individuals aged 65 and older: a systematic review. Journal of the American Geriatrics Society. 2011 Aug;59(8):1521-30.

17. Hanlon JT, Schmader KE, Samsa GP, Weinberger M, Uttech KM, Lewis IK, et al. A method for assessing drug therapy appropriateness. Journal of clinical epidemiology. 1992 Oct;45(10):1045-51.

18. Clyne B, Fitzgerald C, Quinlan A, Hardy C, Galvin R, Fahey T, et al. Interventions to Address Potentially Inappropriate Prescribing in Community-Dwelling Older Adults: A Systematic Review of Randomized Controlled Trials. Journal of the American Geriatrics Society. 2016 Jun;64(6):1210-22.

19. Kaur S, Mitchell G, Vitetta L, Roberts MS. Interventions that can reduce inappropriate prescribing in the elderly: a systematic review. Drugs Aging. 2009;26(12):1013-28.

20. Patterson SM, Cadogan CA, Kerse N, Cardwell CR, Bradley MC, Ryan C, et al. Interventions to improve the appropriate use of polypharmacy for older people. The Cochrane database of systematic reviews. 2014 (10):CD008165.

21. Lainer M, Mann E, Sonnichsen A. Information technology interventions to improve medication safety in primary care: a systematic review. International journal for quality in health care : journal of the International Society for Quality in Health Care. 2013 Oct;25(5):590-8.

22. Randolph AG, Haynes RB, Wyatt JC, Cook DJ, Guyatt GH. Users' Guides to the Medical Literature: XVIII. How to use an article evaluating the clinical impact of a computer-based clinical decision support system. Jama. 1999 Jul 07;282(1):67-74.

23. Soiza RL, Subbarayan S, Antonio C, Cruz-Jentoft AJ, Petrovic M, Gudmundsson A, et al. The SENATOR project: developing and trialling a novel software engine to optimize medications and nonpharmacological therapy in older people with multimorbidity and polypharmacy. Therapeutic advances in drug safety. 2017;8(3):81-5.

24. Young A, Tordoff J, Dovey S, Reith D, Lloyd H, Tilyard M, et al. Using an Electronic Decision Support Tool to Reduce Inappropriate Polypharmacy and Optimize Medicines: Rationale and Methods. JMIR Research Protocols. 2016;5(2):e105.

25. Mangin D, Stephen G, Bismah V, Risdon C. Making patient values visible in healthcare: a systematic review of tools to assess patient treatment priorities and preferences in the context of multimorbidity. BMJ Open. 2016;6(6):e010903.

26. van Summeren JJ, Schuling J, Haaijer-Ruskamp FM, Denig P. Outcome prioritisation tool for medication review in older patients with multimorbidity: a pilot study in general practice. British Journal of General Practice. 2017.

27. Prados-Torres A, Del Cura-Gonzalez I, Prados-Torres D, Lopez-Rodriguez JA, Leiva-Fernandez F, Calderon-Larranaga A, et al. Effectiveness of an intervention for improving drug prescription in primary care patients with multimorbidity and polypharmacy: study protocol of a cluster randomized clinical trial (Multi-PAP project). Implementation science : IS. 2017 Apr 27;12(1):54.

28. Clyne B, Smith SM, Hughes CM, Boland F, Bradley MC, Cooper JA, et al. Effectiveness of a Multifaceted Intervention for Potentially Inappropriate Prescribing in Older Patients in Primary Care: A Cluster-Randomized Controlled Trial (OPTI-SCRIPT Study). Annals of family medicine. 2015 Nov;13(6):545-53.

29. Clyne B, Cooper JA, Hughes CM, Fahey T, Smith SM. A process evaluation of a cluster randomised trial to reduce potentially inappropriate prescribing in older people in primary care (OPTI-SCRIPT study). Trials. 2016;17(1):386.

30. Dreischulte T, Donnan P, Grant A, Hapca A, McCowan C, Guthrie B. Safer Prescribing--A Trial of Education, Informatics, and Financial Incentives. The New England journal of medicine. 2016 Mar 17;374(11):1053-64.

31. Craig P, Dieppe P, Macintyre S, Michie S, Nazareth I, Petticrew M. Developing and evaluating complex interventions: the new Medical Research Council guidance. BMJ. 2008;337:a1655.

32. Reuben DB, Tinetti ME. Goal-oriented patient care--an alternative health outcomes paradigm. The New England journal of medicine. 2012 Mar 01;366(9):777-9.

33. Dreischulte T, Grant AM, McCowan C, McAnaw JJ, Guthrie B. Quality and safety of medication use in primary care: consensus validation of a new set of explicit medication assessment criteria and prioritisation of topics for improvement. BMC clinical pharmacology. 2012;12:5.

34. Howard RL, Avery AJ, Slavenburg S, Royal S, Pipe G, Lucassen P, et al. Which drugs cause preventable admissions to hospital? A systematic review. Br J Clin Pharmacol. 2007 Feb;63(2):136-47.

35. Clyne B, Bradley MC, Hughes CM, Clear D, McDonnell R, Williams D, et al. Addressing potentially inappropriate prescribing in older patients: development and pilot study of an intervention in primary care (the OPTI-SCRIPT study). BMC health services research. 2013;13:307.

36. Todd A, Holmes HM. Recommendations to support deprescribing medications late in life. International journal of clinical pharmacy. 2015;37(5):678-81.

37. Gnjidic D, Le Couteur DG, Kouladjian L, Hilmer SN. Deprescribing Trials: Methods to Reduce Polypharmacy and the Impact on Prescribing and Clinical Outcomes. Clinics in geriatric medicine. 2012;28(2):237-53.

38. Eldridge SM, Lancaster GA, Campbell MJ, Thabane L, Hopewell S, Coleman CL, et al. Defining Feasibility and Pilot Studies in Preparation for Randomised Controlled Trials: Development of a Conceptual Framework. PloS one. 2016;11(3):e0150205.

39. Campbell MK, Elbourne DR, Altman DG. CONSORT statement: extension to cluster randomised trials. Bmj. 2004 Mar 20;328(7441):702-8.

40. Eldridge SM, Ashby D, Feder GS, Rudnicka AR, Ukoumunne OC. Lessons for cluster randomized trials in the twenty-first century: a systematic review of trials in primary care. Clinical trials (London, England). 2004 Feb;1(1):80-90.

41. Brilleman SL, Salisbury C. Comparing measures of multimorbidity to predict outcomes in primary care: a cross sectional study. Family practice. 2013 Apr;30(2):172-8.

42. O'Kelly M, Teljeur C, O'Kelly F, Ni Shúilleabháin A, O'Dowd T. Structure of General Practice in Ireland 1982 — 2015. ICGP: 2016.

43. General Medical Services Scheme. Dublin Department of Public Expenditure and Reform, 2016.

44. Duncan P, Salisbury C, Murphy M, Man M-S, Chaplin K, Mann C, editors. Development and validation of the Multimorbidity Treatment Burden Questionnaire. SAPC ASM; 2017; Warwick.

45. EuroQol--a new facility for the measurement of health-related quality of life. Health policy (Amsterdam, Netherlands). 1990 Dec;16(3):199-208.

46. Reeve E, Low LF, Shakib S, Hilmer SN. Development and Validation of the Revised Patients' Attitudes Towards Deprescribing (rPATD) Questionnaire: Versions for Older Adults and Caregivers. Drugs Aging. 2016 Dec;33(12):913-28.

47. Gerety MB, Cornell JE, Plichta DT, Eimer M. Adverse events related to drugs and drug withdrawal in nursing home residents. Journal of the American Geriatrics Society. 1993 Dec;41(12):1326-32.

48. Graves T, Hanlon JT, Schmader KE, Landsman PB, Samsa GP, Pieper CF, et al. Adverse events after discontinuing medications in elderly outpatients. Arch Intern Med. 1997 Oct 27;157(19):2205-10.

49. Wallace E, McDowell R, Bennett K, Fahey T, Smith SM. Impact of Potentially Inappropriate Prescribing on Adverse Drug Events, Health Related Quality of Life and Emergency Hospital Attendance in Older People Attending General Practice: A Prospective Cohort Study. J Gerontol A Biol Sci Med Sci. 2016 Jul 27.

50. Johansson T, Abuzahra ME, Keller S, Mann E, Faller B, Sommerauer C, et al. Impact of strategies to reduce polypharmacy on clinically relevant endpoints: a systematic review and meta-analysis. Br J Clin Pharmacol. 2016 Aug;82(2):532-48.

51. Grant A, Treweek S, Dreischulte T, Foy R, Guthrie B. Process evaluations for cluster-randomised trials of complex interventions: a proposed framework for design and reporting. Trials. 2013;14(1):1-10.

52. Novick G. Is There a Bias Against Telephone Interviews In Qualitative Research? Research in nursing & health. 2008;31(4):391-8.

53. Guidelines for the Economic Evaluation of Health Technologies in Ireland. HIQA Guideline. 2014.

54. Löffler C, Kaduszkiewicz H, Stolzenbach C-O, Streich W, Fuchs A, van den Bussche H, et al. Coping with multimorbidity in old age – a qualitative study. BMC Family Practice. 2012;13(1):1-8.

55. Noel PH, Frueh BC, Larme AC, Pugh JA. Collaborative care needs and preferences of primary care patients with multimorbidity. Health expectations : an international journal of public participation in health care and health policy. 2005 Mar;8(1):54-63.

56. Tinetti ME, Bogardus STJ, Agostini JV. Potential Pitfalls of Disease-Specific Guidelines for Patients with Multiple Conditions. New England Journal of Medicine. 2004;351(27):2870-4.

57. Glass J, Lanctot KL, Herrmann N, Sproule BA, Busto UE. Sedative hypnotics in older people with insomnia: meta-analysis of risks and benefits. Bmj. 2005 Nov 19;331(7526):1169.

58. Ballard CG, Waite J, Birks J. Atypical antipsychotics for aggression and psychosis in Alzheimer's disease. Cochrane Database of Systematic Reviews. 2006 (1).

*Figure 1: Development and evaluation of SPPiRE, adapted from MRC framework* ([31](#_ENREF_31))

Legend: Adaptation of the MRC framework showing the evolution of the SPPiRE intervention based on previous outcome and process evaluations, piloting and feasibility work and emerging evidence.

*Figure 2: Flow of practices and patients through RCT*

*Figure 3: SPPiRE medication review process*

Abbreviations: *PIP* (Potentially inappropriate prescription, *ADR* (Adverse drug reaction)

*Table 1: Evolution of SPPiRE from OPTI-SCRIPT*

|  | **OPTI-SCRIPT(**[**28**](#_ENREF_28)**)** | **SPPiRE** |
| --- | --- | --- |
| **Population** | Research pharmacist determined if identified older patients had a PIP | Identified by patient finder tool as being prescribed≥ 15 repeat medicines |
| **Intervention** | Academic detailing by research pharmacist | Training video by research GP |
|  | Web-guided medication review  Provided with alternative pharmaceutical treatment algorithms for PIP | Web-guided medication review  Guided to check for specific PIP  Guided to tailor care to patient’s priorities |
| **Primary outcomes** | Proportion of patients with PIP | Proportion of patients with PIP |
|  | Mean number of PIPs per patient | Number of repeat medications |
| **Secondary outcomes** | Drug specific outcomes | Medication changes |
|  | Patient reported outcomes  Quality of life  Patient's medication beliefs  Patient well being | Patient reported outcomes  Quality of life  Patient's attitudes to deprescribing  Treatment burden |

*Table 2: SPPiRE PIP; drug groups frequently implicated in preventable drug related morbidity (*[*34*](#_ENREF_34)*)*

| **Drug group** | **PIP** | **Reason** |
| --- | --- | --- |
| **NSAIDS** | with diuretic **and** ACEi/ARB ([33](#_ENREF_33)) | Risk of renal impairment |
|  | with chronic kidney disease (eGFR <50 ml/min/ 1.73m^2^) ([15](#_ENREF_15), [33](#_ENREF_33)) |  |
|  | for ≥ 12 weeks with no gastroprotection ([33](#_ENREF_33)) | Risk of GI bleed |
|  | with a history of PUD with no gastroprotection ([15](#_ENREF_15)) |  |
|  | and antiplatelet with no gastroprotection ([15](#_ENREF_15)) |  |
|  | with an anticoagulant ([15](#_ENREF_15), [35](#_ENREF_35)) |  |
|  | with severe hypertension or heart failure ([15](#_ENREF_15)) | Risk of hypertension/ heart failure exacerbation |
|  | COX-2 selective with concurrent cardiovascular disease ([15](#_ENREF_15)) | Increased risk of MI/CVA |
| **Antiplatelets** | and history of PUD with no gastroprotection ([33](#_ENREF_33), [35](#_ENREF_35)) | Risk of GI bleed |
|  | and anticoagulant with no gastroprotection ([33](#_ENREF_33), [35](#_ENREF_35)) |  |
|  | aspirin and clopidogrel with no gastroprotection ([33](#_ENREF_33)) |  |
|  | consider intended duration of treatment if taking dual anti-platelet therapy for over one year post PCI ([15](#_ENREF_15)) | Not usually indicated |
| **Anticoagulants** | for first uncomplicated DVT for >6 months duration ([15](#_ENREF_15)) | Not indicated |
|  | for first uncomplicated PE for >12 months duration ([15](#_ENREF_15)) |  |
|  | dabigatran if eGFR <30 ml/min/ 1.73m^2^ or if renal function is unknown ([15](#_ENREF_15)) | Risk of bleeding |
|  | rivaroxaban or apixaban if eGFR <15 ml/min/ 1.73m^2^ or if renal function is unknown ([15](#_ENREF_15)) |  |
| **Diuretics** | and no renal profile in the last 48 weeks ([33](#_ENREF_33)) | Risk of renal impairment and electrolyte abnormality |
|  | loop diuretic and thiazide diuretic and no renal profile in the last 24 weeks ([33](#_ENREF_33)) |  |
|  | loop diuretic for dependent oedema and no heart failure, liver failure or nephrotic syndrome ([15](#_ENREF_15)) | Risks usually out-weigh benefits |
|  | thiazide diuretic with a history of gout ([15](#_ENREF_15)) | Risk of precipitating gout |

Abbreviations: *NSAID* (Non-steroidal anti-inflammatory drug), *ACEi* (Angiotensin converting enzyme inhibitor), *ARB* (Aldosterone receptor blocker), *eGFR* (Estimated glomerular filtration rate), *PUD* (Peptic ulcer disease), *GI* (Gastro-intestinal), *MI* (Myocardial infarction), *CVA* (Cerebrovascular accident), *COX-2* (Cyclooxygenase-2), *DVT* (Deep vein thrombosis), *PCI* (Percutaneous coronary intervention), *PE,* (Pulmonary embolism)

*Table 3: SPPiRE PIP; drug groups associated with morbidity in the elderly (*[*33*](#_ENREF_33)*)*

| **Drug group** | **PIP** | **Reason** |
| --- | --- | --- |
| **Anticholinergic drugs** | With comorbidities ([35](#_ENREF_35))   - Dementia - Narrow angle glaucoma - Cardiac conduction abnormalities - Chronic prostatism | Exacerbation of co-morbidity |
|  | concomitant use of two or more drugs with anticholinergic properties ([15](#_ENREF_15)) | Risk of anticholinergic toxicity |
|  | tricyclic antidepressant as first line antidepressant ([15](#_ENREF_15)) | Increased risk of adverse effects in older patients and alternatives available |
|  | antimuscarinic antihistamine ([15](#_ENREF_15)) |  |
| **Benzodiazipines OR Z drugs** | for longer than 4 weeks ([15](#_ENREF_15)) ([33](#_ENREF_33)) | Risk of sedation, confusion, impaired balance, falls.  NNT 13 and NNH 6 when used for insomnia ([57](#_ENREF_57)) |
| **Antipsychotics** | with dementia and no psychosis ([15](#_ENREF_15), [33](#_ENREF_33)) | Increased risk of stroke, only use when all other means have failed and shortest possible dose for shortest duration ([58](#_ENREF_58)) |

Abbreviations: *NNT* (Number needed to treat), *NNH* (Number needed to harm)

*Table 4: SPPiRE PIP, miscellaneous*

| **Drug group** | **PIP** | **Reason** |
| --- | --- | --- |
| **Methotrexate** | not prescribed as weekly ([33](#_ENREF_33)) | Increased risk of potentially fatal medication errors |
|  | prescribed > 1 strength tablet ([33](#_ENREF_33)) |  |
| **Opioids** | used regularly with no laxative ([15](#_ENREF_15)) | Risk of severe constipation |
| **Corticosteroids** | use ≥ 12 weeks with no bone protection ([15](#_ENREF_15)) | Risk of fracture |
| **PPI** | for uncomplicated PUD/erosive peptic oesophagitis at full therapeutic dose ≥ 8 weeks ([15](#_ENREF_15)) | Not indicated |
| **Metformin** | with eGFR < 30 ml/min/ 1.73m^2^ ([15](#_ENREF_15)) | Risk of lactic acidosis |

Abbreviations: *eGFR* (Estimated glomerular filtration rate), *PUD* (Peptic ulcer disease)

*Table 5: SPPiRE Pilot Results*

| **Pilot Component** | **Problem** | **Change** |
| --- | --- | --- |
| Finder tool | Over identification of patients | - Include only items with unique ATC code - Include only items listed as “current” |
| Educational videos | Not fully watched by all GPs due to time constraint | - Content condensed - Focus on demonstrating review process |
| Web-guided medication review | Not fully completed by all GPs | - Website layout modified - Instructions more explicit - Recommendation to allow for more time |
