# Supplementary material for: GP-delivered medication review of polypharmacy, deprescribing, and patient priorities in older people with multimorbidity in Irish primary care (SPPiRE Study): A cluster randomised controlled trial
Source: PLoS Med. 2022 Jan 5;19(1):e1003862. doi: 10.1371/journal.pmed.1003862 (PMC8730438; doi:10.1371/journal.pmed.1003862)
Supplement: S2 Table — SPPiRE, Supporting Prescribing in Older Adults with Multimorbidity in Irish Primary Care. (DOCX) [file pmed.1003862.s003.docx]

# S2 Table

SPPiRE Criteria

| **Drug group** | **PIP** | **Reason** |  |
| --- | --- | --- | --- |
| **Drug groups frequently associated with preventable drug related morbidity** | | |  |
| **NSAIDS** | with diuretic and ACEi/ARB (1) | Risk of renal impairment |  |
|  | with chronic kidney disease (eGFR <50) (1, 2) |  |  |
|  | for ≥ 12 weeks with no gastroprotection (1) | Risk of GI bleed |  |
|  | that is not COX 2 selective, with a history of PUD with no gastroprotection (2) |  |  |
|  | and antiplatelet with no gastroprotection (2) |  |  |
|  | with an anticoagulant (2, 3) |  |  |
|  | with severe hypertension or heart failure (2) | Risk of hypertension/ heart failure exacerbation |  |
|  | COX-2 selective with concurrent cardiovascular disease (2) | Increased risk of MI/CVA |  |
| **Antiplatelets** | and history of PUD with no gastroprotection (1, 3) | Risk of GI bleed |  |
|  | and anticoagulant with no gastroprotection (1, 3) |  |  |
|  | dual antiplatelet therapy with no gastroprotection (1) |  |  |
|  | consider intended duration of treatment if taking dual anti-platelet therapy for over one year post PCI (2) | Not usually indicated |  |
| **Anticoagulants** | for first uncomplicated DVT for >6 months duration (2) | Not indicated |  |
|  | for first uncomplicated PE for >12 months duration (2) |  |  |
|  | dabigatran (Pradaxa) if eGFR <30 ml/min/ 1.73m^2^ or if renal function is unknown (2) | Risk of bleeding |  |
|  | rivaroxaban (Xarelto)or apixaban (Eliquis) if eGFR <15 ml/min/ 1.73m^2^ or if renal function is unknown (2) |  |  |
| **Diuretics** | and no U&E check in the last 48 weeks (1) | Risk of renal impairment and electrolyte abnormality |  |
|  | loop diuretic and thiazide diuretic and no U&E in the last 24 weeks (1) |  |  |
|  | loop diuretic for dependent oedema and no heart failure, liver failure or nephrotic syndrome (2) | Risks usually out-weigh benefits |  |
|  | thiazide diuretic with a history of gout (2) | Risk of precipitating gout |  |
| **Drugs groups associated with morbidity in the elderly** | | | |
| **Anticholinergic drugs** | With comorbidities (3)  Dementia  Narrow angle glaucoma  Cardiac conduction abnormalities  Chronic prostatism | Exacerbation of co-morbidity | |
|  | Concomitant use of two or more drugs with anticholinergic properties (2) | Risk of anticholinergic toxicity | |
|  | tricyclic antidepressant as first line antidepressant (2) | Increased risk of adverse effects in older patients and alternatives available | |
|  | antimuscarinic antihistamine (2) |  |  |
| **Benzodiazepines OR Z drugs** | for longer than 4 weeks (2) (1) | Risk of sedation, confusion, impaired balance, falls.  NNT 13 and NNH 6 when used for insomnia (4) | |
| **Antipsychotics** | with dementia and no psychosis (1, 2) | Increased risk of stroke, only use when all other means have failed and shortest possible dose for shortest duration (5) | |
| **Miscellaneous drug groups; included because of prevalence or high risk** | | |  |
| **Methotrexate** | not prescribed as weekly (1) | Increased risk of potentially fatal medication errors |  |
|  | prescribed > 1 strength tablet (1) |  |  |
| **Opioids** | used regularly with no laxative (2) | Risk of severe constipation |  |
| **Corticosteroids** | use ≥ 12 weeks with no bone protection (2) | Risk of fracture |  |
| **PPI** | for uncomplicated PUD/erosive peptic oesophagitis at full therapeutic dose ≥ 8 weeks (2) | Not indicated |  |
| **Metformin** | with eGFR < 30 ml/min/ 1.73m^2^ (2) | Risk of lactic acidosis |  |

*Abbreviations: NSAID; non-steroidal anti-inflammatory drug, ACEi; angiotensin converting enzyme inhibitor, ARB; aldosterone receptor blocker, eGFR; estimated glomerular filtration rate, PUD; peptic ulcer disease, GI; gastro-intestinal, MI; myocardial infarction, CVA; cerebrovascular accident, COX-2; cyclooxygenase-2, DVT; deep vein thrombosis, PCI; percutaneous coronary intervention, PE; pulmonary embolism, NNT; number needed to treat, NNH; number needed to harm*

1. Dreischulte T, Grant AM, McCowan C, McAnaw JJ, Guthrie B. Quality and safety of medication use in primary care: consensus validation of a new set of explicit medication assessment criteria and prioritisation of topics for improvement. BMC clinical pharmacology. 2012;12:5.

2. O'Mahony D, O'Sullivan D, Byrne S, O'Connor MN, Ryan C, Gallagher P. STOPP/START criteria for potentially inappropriate prescribing in older people: version 2. Age and ageing. 2015;44(2):213-8.

3. Clyne B, Bradley MC, Hughes CM, Clear D, McDonnell R, Williams D, et al. Addressing potentially inappropriate prescribing in older patients: development and pilot study of an intervention in primary care (the OPTI-SCRIPT study). BMC health services research. 2013;13:307.

4. Glass J, Lanctot KL, Herrmann N, Sproule BA, Busto UE. Sedative hypnotics in older people with insomnia: meta-analysis of risks and benefits. Bmj. 2005;331(7526):1169.

5. Ballard CG, Waite J, Birks J. Atypical antipsychotics for aggression and psychosis in Alzheimer's disease. Cochrane Database of Systematic Reviews. 2006(1).
