# Supplementary material for: GP-delivered medication review of polypharmacy, deprescribing, and patient priorities in older people with multimorbidity in Irish primary care (SPPiRE Study): A cluster randomised controlled trial
Source: PLoS Med. 2022 Jan 5;19(1):e1003862. doi: 10.1371/journal.pmed.1003862 (PMC8730438; doi:10.1371/journal.pmed.1003862)
Supplement: S3 Table — (DOCX) [file pmed.1003862.s004.docx]

# S3 Table

Comparison of participants lost to follow up for both primary outcomes and patient reported secondary outcomes

Table 1, Comparison of all participants with those lost to follow up and followed up for primary outcomes

| **Characteristic** | **All participants** | | **Lost to follow up** | | **Followed up** | |
| --- | --- | --- | --- | --- | --- | --- |
|  | **I (N=208)** | **C (N=196)** | **I (N=16)** | **C (N=19)** | **I (N=192)** | **C (N=177)** |
| Mean age (SD) | 76.67 (6.80) | 76.33 (6.88) | 81.93 (7.57) | 79.78 (7.35) | 76.24 (6.57) | 75.95 (6.74) |
| % Female | 57.21 | 57.14 | 37.50 | 78.95 | 58.85 | 54.80 |
| Mean number medicines at baseline (SD) | 16.96 (3.25) | 17.82 (3.71) | 18.06 (3.99) | 18.74 (4.82) | 16.87 (3.17) | 17.72 (3.57) |
| Mean PIP baseline (SD) | 2.50  (1.53) | 2.53  (1.40) | 2.69  (1.49) | 2.63  (1.64) | 2.47  (1.52) | 2.55  (1.43) |
| % with ≥ 1 PIP | 93.24 | 92.82 | 93.75 | 93.18 | 93.19 | 88.69 |
| Mean EQVAS (SD) | 59.63 (20.24) | 59.75 (22.09) | 48.67 (15.75) | 58.42 (19.44) | 60.54 (20.34) | 59.90 (22.45) |
| % with GMS card | 82.00 | 89.78 | 73.33 | 100.00 | 82.70 | 88.69 |

*Abbreviations: I; intervention, C; control, SD; standard deviation, PIP; potentially inappropriate prescription, EQVAS; EQ – 5D visual analogue scale, GMS; general medical services.*

Table 2, Comparison of participants with and without follow up PROM data

| **Characteristic** | **PROM data at follow up**  **n=223 (55%)** | **No PROM data at follow up**  **n=181 (45%)** |
| --- | --- | --- |
| Male (%)  Female (%) | 110 (49.3)  113 (50.7) | 63 (34.8)  118 (65.2) |
| SEG 1 or 2 (%)  Other SEG (%) | 47 (21.1)  175 (78.5) | 40 (22.1)  139 (76.8) |
| Mean age (SD) | 75.86 (6.57) | 77.30 (7.08) |
| Mean no. meds (SD) | 17.35 (3.35) | 17.40 (3.69) |
| Mean no. PIP (SD) | 2.49 (1.41) | 2.56 (1.57) |

*Abbreviations: SD; standard deviation, PIP; potentially inappropriate prescription, SEG: socio-economic group.*
