# Supplementary material for: GP-delivered medication review of polypharmacy, deprescribing, and patient priorities in older people with multimorbidity in Irish primary care (SPPiRE Study): A cluster randomised controlled trial
Source: PLoS Med. 2022 Jan 5;19(1):e1003862. doi: 10.1371/journal.pmed.1003862 (PMC8730438; doi:10.1371/journal.pmed.1003862)
Supplement: S4 Table — (DOCX) [file pmed.1003862.s005.docx]

# S4 Table

Secondary outcome measures at baseline by treatment group

| **Outcome measure** | **Intervention (n=208)** | **Control (n=196)** |
| --- | --- | --- |
| **Secondary outcome measures** | | |
| **Prescribing measures** | | |
| **Number of PIP (N)**  Mean (SD)  Median (IQR) | N=208  2.49 (1.52)  2 (1 – 3) | N=196  2.57 (1.45)  3 (2 – 3) |
| **Proportion of patients with at least 1 high risk PIP (N)**  N (%) | N=202  124 (61.39) | N=192  130 (67.71) |
| **Patient reported measures** | | |
| **EQ – 5D – 5L index score (N)**  Mean (SD)  **EQ – 5D – 5L VAS (N)**  Mean (SD) | 189  0.50 (0.36)  196  59.63 (20.25) | 184  0.47 (0.38)  185  59.75 (22.09) |
| **MTBQ global score (N)**  Median (IQR) | 199  9.62 (2.27 – 17.31) | 184  9.62 (3.85 – 23.08) |
| **rPATD scores**  Involvement Median (IQR), N  Burden Median (IQR), N  Appropriateness Median (IQR), N  Concern about stopping Median (IQR), N | 4.0 (3.8 – 4.6), 178  2.8 (2.4 – 3.4), 184  3.2 (2.6 – 3.8), 187  2.6 (2.0 – 3.0), 191 | 4.2 (38. – 4.8), 191  3.0 (2.6 – 3.6), 171  3.0 (2.4 – 3.8), 173  2.8 (2.2 – 3.4), 172 |
| **Health care utilisation** | | |
| GP visits Mean (SD), N  Repeat prescriptions Mean (SD), N  ED presentations Mean (SD), N  Hospital admissions Mean (SD), N | 4.81 (3.67), 194  2.69 (2.93), 194  0.38 (0.77), 194  0.34 (0.65), 194 | 4.49 (3.12), 177  2.88 (3.56), 177  0.31 (0.62), 177  0.33 (0.61), 171 |

*Abbreviations: PIP; potentially inappropriate prescription, SD; standard deviation, IQR; interquartile range, EQ -5D - 5L; EuroQoL 5-dimension 5-level, MTBQ; Multimorbidity treatment burden questionnaire, rPATD; revised patients’ attitudes towards deprescribing, ED; emergency department*
