# Supplementary material for: GP-delivered medication review of polypharmacy, deprescribing, and patient priorities in older people with multimorbidity in Irish primary care (SPPiRE Study): A cluster randomised controlled trial
Source: PLoS Med. 2022 Jan 5;19(1):e1003862. doi: 10.1371/journal.pmed.1003862 (PMC8730438; doi:10.1371/journal.pmed.1003862)
Supplement: S5 Table — (DOCX) [file pmed.1003862.s006.docx]

# S5 Table Sensitivity analyses

| ***Analysis of primary outcome, number of medicines at follow up, using mixed Poisson regression*** | | | | | | |
| --- | --- | --- | --- | --- | --- | --- |
| Description | Included | Excluded | MCS | IRR | p value | 95% CI |
| ITT (all participants) | 404 | 0 | 7.9 | 0.95 | 0.045 | 0.899 – 0.999 |
| CCA (all participants with follow up data) ^a^ | 369 | 35 | 7.4 | 0.94 | 0.044 | 0.883 – 0.999 |
| PPA (only participants who had intervention and have follow up data) ^a^ | 332 | 72 | 7.1 | 0.97 | 0.115 | 0.943 – 1.006 |
| ITT including number of days to follow up as covariate ^b^ | 404 | 0 | 7.9 | 0.95 | 0.099 | 0.902 – 1.008 |
| ITT including presence of a repeat prescribing policy as a covariate ^a^ | 404 | 0 | 7.9 | 0.95 | 0.099 | 0.902 – 1.008 |
| Sensitivity analysis excluding GP116 (median number of medicines <14) ^c^ | 389 | 15 | 7.8 | 0.94 | 0.044 | 0.893 – 0.999 |
| PPA (only participants who had intervention and have follow up data and excluding 3 control practices with a reduction in mean no meds>3) ^d^ | 314 | 90 | 7.1 | 0.96 | 0.008 | 0.936 – 0.990 |
| COVID follow up (including whether participant was followed up after March 2020 as a covariate) ^e^ | 404 | 0 | 7.9 | 0.95 | 0.45 | 0.895 – 0.999 |
| ***Analysis of primary outcome, patients with at least one PIP, using logistic regression*** | | | | | | |
| Description | Included | Excluded | MCS | OR | p value | 95% CI |
| ITT (all participants) | 402 | 2 | 7.9 | 0.39 | 0.066 | 0.140 – 1.064 |
| CCA (all participants with follow up data) ^a^ | 367 | 37 | 7.3 | 0.39 | 0.066 | 0.141 – 1.064 |
| PPA (only participants who had intervention and have follow up data) ^a^ | 332 | 72 | 7.1 | 0.97 | 0.115 | 0.943 – 1.006 |
| ITT including number of days to follow up as covariate ^b^ | 402 | 2 | 7.9 | 0.48 | 0.166 | 0.169 – 1.358 |
| ITT including presence of a repeat prescribing policy as a covariate ^a^ | 402 | 2 | 7.9 | 0.37 | 0.048 | 0.135 – 0.991 |
| Sensitivity analysis excluding GP116 (median number of medicines <14) ^c^ | 387 | 17 | 7.7 | 0.38 | 0.069 | 0.133 – 1.079 |
| PPA (only participants who had intervention and have follow up data and excluding 3 control practices with a reduction in mean number meds>3) ^d^ | 313 | 91 | 7.1 | 0.63 | 0.069 | 0.378 – 1.038 |
| COVID follow up (including whether participant was followed up after March 2020 as a covariate) ^e^ | 402 | 2 | 7.9 | 0.37 | 0.052 | 0.139 – 1.008 |

*Abbreviations: MCS; mean cluster size, IRR; incidence rate ration, CI; confidence interval, ITT; intention to treat, CCA; complete case analysis, PPA; per protocol analysis, OR; odds ratio*

^a^ Pre-planned sensitivity analysis

^b^ Un-planned sensitivity analysis due to identified discrepancy in the median number of days to follow up between groups. Due to the process of sequential allocation with minimization, intervention and control practices were not paired in a 1:1 fashion. Delays in intervention implementation in some intervention practices led a chance imbalance in the number of days to follow up between groups.

^c^ Un-planned sensitivity analysis as this practice was noted to recruit several participants prescribed <15medicines once baseline data was complete

^d^ Un-planned sensitivity analysis, three control practices identified that had a very large reduction in the number of medicines per participant

^e^ Un-planned sensitivity analysis to assess the effect of COVID 19 on primary outcomes
