# Supplementary material for: GP-delivered medication review of polypharmacy, deprescribing, and patient priorities in older people with multimorbidity in Irish primary care (SPPiRE Study): A cluster randomised controlled trial
Source: PLoS Med. 2022 Jan 5;19(1):e1003862. doi: 10.1371/journal.pmed.1003862 (PMC8730438; doi:10.1371/journal.pmed.1003862)
Supplement: Attachment — Submitted filename: Response to reviewers and editors (2).docx [file pmed.1003862.s007.docx]

HRB Centre for Primary Care Research

Department of General Practice

Royal College of Surgeons in Ireland

Dublin 2

18/08/2021

Dear Editors and Reviewers,

Thank you for your feedback and comments. We have outlined our responses to each comment with excerpts from the amended manuscript where relevant underneath each comment below.

Best Wishes,

Caroline McCarthy on behalf of all co-authors

**Requests from the editors:**

1.      Abstract:
a)      Please ensure that all numbers presented in the abstract are present and identical to numbers presented in the main manuscript text.

Line 315 of the Adverse Events sub-section of the results has been updated to include the denominator of the number of drugs stopped in the intervention group.

*“Fifteen adverse drug withdrawal events out of 826 stopped drugs in intervention patients (1.81%) were reported by intervention group GPs”*

Line 41 Typographical error in the SD presented is corrected.

b)      Please provide the number of GP practices in each group (i.e., control and intervention groups)
c)      Please indicates the dates during which study enrollment and follow up occurred.

These details have been added into the abstract (lines 32 – 36).

d)      Please include a summary of adverse events in the study.
e)      In the last sentence of the Abstract Methods and Findings section, please describe the main limitation(s) of the study's methodology.

These details have been updated as follows:

*“Adverse events recorded included mortality, emergency department presentations and adverse drug withdrawal events (ADWEs). There was no difference in mortality and ED presentations between groups and less than 2% of drug withdrawals in the intervention group led to a reported ADWE. Due to an inability to electronically extract data, primary outcomes were measured at just two time points and this is the main limitation of this work.”*

2.      Please include the study protocol document and analysis plan, with any amendments, as Supporting Information to be published with the manuscript if accepted.

The study protocol which was submitted for publication prospectively (published July 2017), contains a plan for analysis and is now uploaded as supporting information.

3.      Please merge the Aims section (line #111-115) with the Introduction section. Please conclude the Introduction with a clear description of the study question or hypothesis.

The Aims section has been merged as the final paragraph of the introduction.

4.      In the Methods section’s text, please indicate the number of GP practices in each arm.

Line 152, Randomisation and masking.

*“Recruited practices were allocated to intervention (26) or control groups (25) by minimisation using Minimpy software...”*

5.      When completing the CONSORT checklist, please use section and paragraph numbers.

We have updated the CONSORT checklist to include section and paragraph numbers as suggested.

6.      The terms gender and sex are not interchangeable please use the appropriate term.

The manuscript has been updated and the term sex has been used instead of gender throughout.

7.      Please replace "Social class" with "occupation" or similar throughout the paper.

The manuscript has been updated to use the term occupation instead of social class throughout.

8.      Please use the PLOS Medicine style reference call outs throughout the text, noting the absence of spaces within the square brackets, e.g., "... quality of life [1-3]."

This has been amended throughout the manuscript.


**Comments from the reviewers:**

**We have highlighted the key points here and responses are outlined below.**

**Reviewer #1:** The authors are to be congratulated on completing this cluster RCT in primary care - such trials are hard to do logistically and take a lot of effort. As others have found, including myself, the number of recruitable GP practices (only about a third) and the numbers of recruited patients (about a quarter of those screened) are always less than one hopes at the outset and clearly introduces the potential for selection bias and loss of generalisability, and attenuates the intervention effect as it is likely that GPs more interested in deprescribing specifically and research more generally may express interest in participating but whose practices are already at a more optimal level than those who do not. Similarly control patients who consented to participation, may be more open to medication review and deprescribing, coupled with the inevitable Hawthorne effect in control practices, as was seen in this study (and ours). The fact that about 20% of intervention effects did not receive the medication review, because of limited GP time or patient frailty/illness/death did not help either. The one-off nature of the intervention is a further limitation - **could the authors make further comment about whether any subsequent GP consultations involved any further discussions around deprescribing**. I note further process and qualitative studies are in progress but it would be interesting to see how easy GPs regarded the interaction with the SPPiRE website. While the STOPP criteria are validated they may be difficult to apply in individual cases because no set of rules will cover all possible scenarios, but also they are a limited set of rules and may not cover all potential deprescribing scenarios. I find it intriguing that intervention GPs who chose not to refer to the template but still undertook a deprescribing consultation achieved greater reduction in medications than those who did use the template and this may be explained by the former GPs internalising deprescribing concepts and applying these more generically to more drugs. This may in the end be a more effective approach than GPs referring to, and perhaps being constrained by, a pre-specified drug-class specific template and deserves further study. The loss of **45% of patients reporting PROMs is a further limitation** and again introduces selection bias unless the authors can include as an appendix a comparison of characteristics of patients who did or did not report PROMs. But these limitations do not detract from the basic message that other similar studies have shown (including our own, which the authors may want to reference: Anderson K et al J Am Geriatr Soc 2020: 68:403-410), that deprescribing is not easy to do in time-pressured general practice, that the reductions in number of medication at the individual patient level are marginal but still worthwhile and do not cause harm, that follow-up consultations and longer follow-up may realise greater effects, and that remuneration policies for GPs specific to deprescribing or medication reviews are necessary if these tasks are to be performed adequately.
Minor point - **I would put table 2 as the first table because baseline characteristics of both groups ideally come first and place current table 1 as an appendix** as I found it distracting and does not add much to what current table 2 shows. Also in **appendix 4, it was unclear to me what the two sets of sensitivity analyses related to as they have identical headings** and the same column variables although the data in the cells are slightly different and the IRRs are very different so the headings need further elaboration or at least a footnote.

Thank you for your detailed review, comments and suggestions. We agree it is interesting that the intervention GPs who did not adhere to the template achieved greater reductions in the number of medicines. Qualitative data for the trial’s parallel process evaluation is currently being analysed and will hopefully shed further light on the mechanism of action of the intervention. We have addressed your queries and suggestions on a point by point basis below:

**1) Comment on whether any subsequent GP consultations involved any further discussions around deprescribing.**

There was no difference in health care utilisation between treatment groups, suggesting that there were no ‘additional’ deprescribing consultations, though this activity could have been incorporated in routine follow up consultations once the process had been started during the SPPiRE review. The effect of the intervention on routine and subsequent GP care was explored in the semi structured GP interviews that are being analysed as part of the trial’s parallel process evaluation.

**2) The loss of 45% of patients reporting PROMs is a further limitation**

We agree. We believe this reflects the nature of the patient population and have included this as a limitation in the discussion. A table has been added to Appendix 3 that describes the characteristics of those with and without follow-up PROM data.

**3) Move table 1 to an Appendix**

Table 1 is now listed as Appendix 3.

**4) In** **appendix 4, it was unclear to me what the two sets of sensitivity analyses related to as they have identical headings**

Thank you for pointing this out. The second heading for the second set of sensitivity analyses, “Analysis of primary outcome, patients with at least one PIP, using logistic regression” has been added in. See Appendix 5.

**Reviewer #2:** This cluster randomised controlled trial (SPPiRE) conducted in the Republic of Ireland aims to investigate the effect of a GP-delivered, individualised medication review in reducing polypharmacy and potentially inappropriate prescribing in older patients with multimorbidity in primary care.

Comments:

**Can the authors please provide a copy of the study protocol in the supplementary material?** The authors have appropriately acknowledged where the original protocol was not adhered to, and transparently discussed the reasons for deviation within the manuscript.
This includes a change in data collection methods and an updated sample size calculation.
Furthermore, the authors clearly and commendably provide a Protocol Adherence section, with appropriate sensitivity analyses and associated acknowledgement in the study limitations. The CONSORT checklist has been suitably provided in the supplementary material. The authors demonstrate rigor in the randomisation approach conducted and their attempts to minimise bias throughout the study design. Appropriate sensitivity analyses have been completed, including a complete case analysis and a per protocol analysis, which help to demonstrate the robustness of the study findings. A valid modelling methodology has been applied for the study design, data structure, and research question in hand. Overall, this is a well written and clearly presented study, with accurately and fairly communicated study outcomes, and adequately explored study limitations in the discussion section. A small note: "All 404 patients were included in the intention to treat analysis of primary outcome measures, **see 277 Error! Reference source not found…" to be rectified** under the Primary and Secondary Outcomes section.

Thank you for your detailed review and comments. The study protocol which was submitted for publication prospectively (published July 2017), is now uploaded as supporting information. The error under the Primary and Secondary Outcomes section has been fixed.

**Reviewer #3:** Thank you for the opportunity to review this manuscript. The SPPiRE study presents the results of a cluster randomised controlled trial of a GP delivered medication review where the intervention GPs received a web-based education module and template for medication review. The target population was patients aged 65 years or older and with what the authors define as "complex multimorbidity, defined as being prescribed ≥15 regular medicines".
The manuscript is well written, of interest to a wide readership, and the study itself is well conducted and reported.
I do have some comments and thoughts for the authors to consider and address:
**1. The definition of polypharmacy in this study is not one that is widely accepted. Can you please reference why polypharmacy was defined as 15 or more medications? Polypharmacy is most commonly defined as 5 or more medications, and the other common term is hyperpolypharmacy referring to 10 or more medications. Need to please reference your polypharmacy definition of 15 or more meds. Has implications for whether this was an appropriate target outcome.**

The trial management committee decided on the inclusion criteria of 15 or more medicines based on several factors including recommendations from the then recently published NICE multimorbidity guidelines that specifically recommended considering those on 15 or more medicines as being suitable for a multimorbidity approach to care. It was also based on feasibility issues. Based on the national prescribing database, approx. 5% of patients over 65 was using 15 medicines, whereas this was about 20% for those taking 10 or more medicines. We therefore chose 15 or more medicines as a cut-off point as this meant the average sized Irish GP practice would identify a reasonable number of patients and not need to randomly sample their population. We have further clarified this in the paragraph 3 of the Introduction (line 112) as follows:

*“Medication count is a simple objective measure that has been identified as a good proxy measure for multimorbidity, predicting both primary health care usage and mortality [1], and although treatment for a single condition could reach or exceed 5 medications, ≥15 repeat medicines is likely highly specific to multimorbidity. Based on recommendations from the* [*UK NICE multimorbidity guidelines*](https://www.nice.org.uk/guidance/ng56)*, which recommends targeting patients on 15 or more medicines for a multimorbidity approach to care and in keeping with the fact that higher levels of polypharmacy are more likely to be associated with adverse outcomes for patients with multimorbidity, the target population was patients aged ≥65 years, prescribed ≥15 repeat medicines [2].”*

**2. Polypharmacy and multimorbidity are not the same. Why was multimorbidity defined using the criteria of polypharmacy (15 or more meds)?
Further, patients may have one medical disease e.g. heart failure and be on 5 medications for its treatment so has implications for your study treatment effect. Please comment**.

Thank you for this comment. We agree polypharmacy and multimorbidity are not synonymous however both are linked and medication count has been identified as a suitable proxy measure for multimorbidity. It had the added advantage of being an objective measure that was relatively easy for participating GPs to use as a selection criteria. Furthermore, a software finder tool was developed that could search the electronic care record for numbers of medicines, thus avoiding the challenge of identifying multiple conditions when universal condition coding does not apply. We have elaborated on this in line 112, paragraph 3 of the introduction, see comment above.

*“Medication count is a simple, objective measure that has been identified as a good proxy measure for multimorbidity, predicting both primary health care usage and mortality [1], and although treatment for a single condition could reach or exceed 5 medications, ≥15 repeat medicines is likely highly specific to multimorbidity.”*

**3. Would also suggest avoiding arbitrary or emotive adjectives such as "extreme level of polypharmacy" (line 84) or "significant" polypharmacy (line 175) when talking about an outcome measure or inclusion criteria. Ok to use such words in narrative writing such as in the intro etc. Unless of course this is a formal measure that you can reference. But no citations have been provided as per comment 1 above.**

We agree. The term extreme has been removed throughout the manuscript. A recent systematic review has demonstrated that a range of terms have been used for different degrees of polypharmacy based on numerical definitions [3]. There is no established term to describe 15 or more medicines. Our research group used the term “significant polypharmacy” to refer to our target population prescribed 15 or more repeat medicines. We used this term in the title of our research and have referred to significant polypharmacy as being 15 or more medicines throughout previous SPPiRE publications (the trial protocol, process evaluation protocol and intervention development paper). We only use this term to characterise the degree of polypharmacy and it is not a reported outcome measure.

**4. Could you please provide a definition of repeat medication (line 227)?**

This is now defined in the Methods section, in the subsection “Study design and participants”, line 139.

*“A repeat medicine was defined as any unique item with a World Health Organisation Anatomical Therapeutic Chemical code on the patient’s current repeat prescription, i.e. indicated by their GP as being an ongoing treatment, for ease of issuing further prescriptions within the patient record.”*

**5. The elephant in the room is COVID-19 - worth some discussion about how you think COVID-19 affected your study.**

The COVID 19 pandemic was declared in Ireland on 12^th^ March 2020. At this stage only one of the 26 intervention practices had yet to complete four of their five SPPIRE medication reviews. These four participants had medications reviews over the telephone with their GP (as opposed to face to face). The implementation of the intervention in this practice will be explored as part of the process evaluation using qualitative data from semi structured interviews with this GP and their participating patients.

We did explore the effect of COVID 19 on our results. We did not include this in the original manuscript for the purpose of brevity. One hundred and six of the 404 participants were followed up after March 2020, when this is added as a covariate in a sensitivity analysis there was no effect on the results. However with respect to health care utilisation, unsurprisingly there were significantly less GP visits and more telephone consultations in the group followed up after March 2020. We have included the results of this sensitivity analysis in Appendix 5 and added an explanation of the effect of COVID on healthcare utilisation to Results, Primary and Secondary outcomes (line 310), as follows:

*“Over a quarter of SPPiRE participants had follow up dates after the outbreak of the COVID-19 pandemic in March 2020, and unsurprisingly this group had significantly less face to face GP visits and significantly more telephone consultations.”*

**6. Do you have data to talk about which PIPs you impacted on? The evidence for deprescribing is stronger for specific classes of medications.**

This is an interesting observation. Our primary outcome was a composite measure of 34 PIP criterion, and the trial was not powered to detect a change in individual PIPs. PIP involving higher risk drugs such as anticoagulants, antiplatelets and non-steroidal anti-inflammatory drugs (NSAID) were of low prevalence, even when looking at a composite measure of all PIP involving those drug classes. For example 12 participants in each group had at least one NSAID PIP at baseline reducing to 10 in the control and 7 in the intervention group at follow up. Overall, there was a higher reduction in all PIP in the intervention compared to control group apart from benzodiazepines (which reduced in the control and remained the same in the intervention group). Due to the small numbers with each individual PIP and the lack of an effect on the overall composite primary outcome measure it is difficult to draw any robust conclusions with respect to the effect of the intervention on PIP and we have not amended the manuscript given these uncertainties.

**7. I felt the Conclusion was introducing new concepts that were not presented in the Discussion. The discussion talks about reasons for the study intervention not being effective included its less intensive nature, but the Conclusion talks about the complexity of patients as being the potential reason for no effect. Can you tie these together?**

To address this comment, an additional sentence has been added to the “Comparison with similar medicines management interventions” (line 399) subsection as follows:

*“In addition patients recruited to the SPPiRE trial were on average older and with a notably higher mean number of medicines.”*

**8. Given your definition of multimorbidity was actually polypharmacy of 15 or meds. Can you compare your study to other trials that have targeted hyperpolypharmacy?**

Two systematic reviews that specifically looked at interventions to reduce polypharmacy included a wide range of studies, however the mean number of medicines was lower than SPPiRE (8.9 [4] and 7.4 [5]respectively, compared to 17.37 in SPPiRE). The interventions employed in most of these studies was pharmaceutical care that was typically provided by either the researcher or pharmacist and fed back to the original prescriber. We felt multimorbidity studies that incorporated a medicines management approach were more similar to the SPPiRE intervention as they included an assessment of patient priorities, and specifically addressed medication use in the context of multimorbidity, and this is why these studies are referred to in the discussion. One of the polypharmacy systematic reviews looked at the number of medicines as an outcome measure and a meta-analysis of the 25 included RCTs showed a small but significant reduction in the mean number of medicines, similar to SPPiRE [5].

The text in paragraph 2 in the “Comparison with other studies” subsection of the discussion (line 419) has been updated to include these two systematic reviews as follows:

*“With respect to polypharmacy studies, similar to SPPiRE, a systemic review looking at interventions to improve the appropriate use of polypharmacy in older people concluded that there was no consistent effect on PIP across the 32 included studies [4]. A meta-analysis of 25 deprescribing RCTs where the mean number of medicines at baseline was 7.4, showed a small but significant reduction in the mean number of medicines [5].”*

**9. Line 155 "discuss and agree changes" - is there a word missing before changes?**

Line 165 (formerly 155) in the Procedures subsection of the methods has been changed to, “*discuss and agree upon any changes with their patient…”*

**10. To reduce word count and repetitiveness the first few paragraphs of the Discussion as well the Strengths and limitations is all focussed on trying to explain your results - this could be truncated to then include comparisons with other relevant studies as per Comment 8.**

The first two sections have been truncated and the comparison section expanded (see responses above).

**Reviewer #4:** Thank you for this article on a very important topic for older adults facing multimorbidity and polypharmacy. My comments are relatively minor but hope to address the international audience and also the multifaceted nature of the polypharmacy problem.
**Line 24: What does SPPiRE stand for? I know that this isn't the initial work coming from this intervention but if you could define the acronym perhaps in the abstract this would help frame the idea of the activity more clearly.**

Thank you. The acronym has now been defined on first use in the background of the abstract.

*“The Supporting Prescribing in Older Adults with Multimorbidity in Irish Primary Care (SPPiRE) trial aimed to investigate the effect of a GP-delivered, individualised medication review in reducing polypharmacy and potentially inappropriate prescribing in older patients with multimorbidity in primary care.”*

**Line 25: If this is an international audience then at some point also spelling out GP (which I am assuming is a general practitioner, or a primary care provider) would make sense.**

This has also now been defined on first use, line 26 of the abstract.

**Line 80-86: I appreciate your description of necessary polypharmacy and your discussion of quality of prescriptions since I feel that polypharmacy is not a "black and white" issue and that several studies treat it as such and focus on numbers rather than individualized treatment plans.**

Thank you.

**Line 135: given that these are community dwelling older adults based on your exclusion criteria you might mention that earlier in the manuscript, as homebound or nursing home patients might be more prone to polypharmacy and this study does not focus on them.**

This detail has now been added into the background of the abstract.

**Line 144: thank you for discussing the issues in blinding in this particular study and your attempts to blind the outcome measures.**
**Line 149: Was there a way to assess whether intervention GPs actually played or watched the videos? Were logins tracked or some way to measure that the intervention was being deployed?**

Whether intervention GPs actually watched the videos was not tracked. Engagement with the educational material was explored in the semi-structured interviews which will be analysed as part of the trial’s parallel process evaluation.

**Line 188: Given the deviation in data collection with the medical history do you think that the lack of standardization of the medical history might have impacted the discussion of necessary polypharmacy? Was there a process that the study manager tried to standardize these data that they received from the practices?**

Yes. A standardised data collection form was developed which was piloted with GPs not involved in the study. Until recently there was no formal chronic disease management programme in Irish primary care so practices varied in their coding of chronic disease. This lack of coding may have influenced the interpretation of PIPs that depend on clinical information and led to an underestimation of the PIP “prescription of a drug with anticholinergic potency with a co-morbidity” and an over estimation of the PIP “use of a loop diuretic for dependent oedema in the absence of heart failure”. This same problem would have arisen if a researcher was collecting the data.

**Line 346: Interesting discussion of the possible Hawthorne effect having some bearing on the study.
Line 350-356: I think that this would be a very good study to undertake, as I had some concerns about the "independent study" nature of the intervention and whether the GPs were able to view and incorporate the material fully. This might also help refine the curriculum to the most high yield points (especially since it was noted that a limiting factor in participation was time constraints) In general I would like to see more discussion about the medical issues between groups and if the polypharmacy being addressed was deemed unnecessary or necessary (and if this was not able to be measured to have it be discussed in limitations, perhaps, since it is discussed in the introduction and quality of prescribing is also addressed in the conclusion)**

Only specific medical information to allow assessment of PIP was collected, general medical information such as the number and type of medical conditions was not collected. Prescription data at both time points was collected for all participants. There were 7,051 repeat medicines prescribed at baseline, of which 1,203 (17.0%) were PIP and 6,779 at follow-up of which 1,054 (15.5%) were PIP. The outcomes analysed for the trial were the pre specified primary and secondary prescribing related outcome measures. The SPPiRE PIP were included based on their prevalence in Irish primary care with a focus on higher risk PIP (which was a conclusion from the precursor OPTI-SCRIPT trial). Aside from assessing whether these SPPIRE PIP were present no other assessment was made as to whether medicines were necessary or not. Our research group is currently undertaking a more detailed analysis of baseline prescriptions, including a description of the prevalence of prescriptions with at least one drug of known low clinical effectiveness. We have further elaborated on the limitations of our outcome measures, which were selected based on the results of the OPTI-SCRIPT trial evaluations and to assess the effect of the intervention. The rationale for the selection of these measures is further described in the SPPiRE intervention development paper [6]. We have added the following to line 381 (Strengths and limitations), as follows:

*“The rationale for the selection of the SPPiRE primary outcome measures is described in detail elsewhere [6], but one limitation of using medication count and explicit measures of medication appropriateness as outcome measures is that there was no assessment of the overall appropriateness of prescriptions.”*

References

1.	Brilleman SL, Salisbury C. Comparing measures of multimorbidity to predict outcomes in primary care: a cross sectional study. Family practice. 2013;30(2):172-8. Epub 2012/10/10. doi: 10.1093/fampra/cms060. PubMed PMID: 23045354; PubMed Central PMCID: PMCPMC3604888.

2.	Multimorbidity: assessment, prioritisation and management of care for people with commonly occurring multimorbidity. NICE Guideline. 2016.

3.	Masnoon N, Shakib S, Kalisch-Ellett L, Caughey GE. What is polypharmacy? A systematic review of definitions. BMC Geriatr. 2017;17(1):230. doi: 10.1186/s12877-017-0621-2.

4.	Rankin A, Cadogan CA, Patterson SM, Kerse N, Cardwell CR, Bradley MC, et al. Interventions to improve the appropriate use of polypharmacy for older people. Cochrane Database Syst Rev. 2018;9(9):CD008165-CD. doi: 10.1002/14651858.CD008165.pub4. PubMed PMID: 30175841.

5.	Johansson T, Abuzahra ME, Keller S, Mann E, Faller B, Sommerauer C, et al. Impact of strategies to reduce polypharmacy on clinically relevant endpoints: a systematic review and meta-analysis. Br J Clin Pharmacol. 2016;82(2):532-48. Epub 2016/04/10. doi: 10.1111/bcp.12959. PubMed PMID: 27059768; PubMed Central PMCID: PMCPMC4972170.

6.	McCarthy C, Moriarty F, Wallace E, Smith SM. The evolution of an evidence based intervention designed to improve prescribing and reduce polypharmacy in older people with multimorbidity and significant polypharmacy in primary care (SPPiRE). Journal of Comorbidity. 2020;10:2235042X20946243. doi: 10.1177/2235042X20946243.
