# Supplementary material for: GP-delivered medication review of polypharmacy, deprescribing, and patient priorities in older people with multimorbidity in Irish primary care (SPPiRE Study): A cluster randomised controlled trial
Source: PLoS Med. 2022 Jan 5;19(1):e1003862. doi: 10.1371/journal.pmed.1003862 (PMC8730438; doi:10.1371/journal.pmed.1003862)
Supplement: Attachment — Submitted filename: Response to editors 03.11.2021.docx [file pmed.1003862.s008.docx]

HRB Centre for Primary Care Research

Department of General Practice

Royal College of Surgeons in Ireland

Dublin 2

03/11/2021

Dear Editors and Reviewers,

Thank you for reviewing our revised manuscript. We have amended our title as follows, “GP-delivered medication review of polypharmacy, deprescribing and patient priorities in older people with multimorbidity in Irish primary care (SPPiRE study): a cluster randomised controlled trial”.

Best Wishes,

Caroline McCarthy on behalf of all co-authors
